# Yeast-2-Hybrid data file showing progranulin interactions in human fetal brain and bone marrow libraries

**DOI:** 10.1016/j.dib.2016.11.031

**Published:** 2016-11-19

**Authors:** Irmgard Tegeder

**Affiliations:** Institute of Clinical Pharmacology, Goethe-University Hospital, Frankfurt, Germany

**Keywords:** Yeast-2-Hybrid, Protein interactions, Gene ontology, Progranulin

## Abstract

Progranulin deficiency in humans is associated with neurodegeneration. Its mechanisms are not yet fully understood. We performed a Yeast-2-Hybrid screen using human full-length progranulin as bait to assess the interactions of progranulin. Progranulin was screened against human fetal brain and human bone marrow libraries using the standard Matchmaker technology (Clontech). This article contains the full Y2H data table, including blast results and sequences, a sorted table according to selection criteria for likely positive, putatively positive, likely false and false preys, and tables showing the gene ontology terms associated with the likely and putative preys of the brain and bone marrow libraries. The interactions with autophagy proteins were confirmed and functionally analyzed in "Progranulin overexpression in sensory neurons attenuates neuropathic pain in mice: Role of autophagy" (C. Altmann, S. Hardt, C. Fischer, J. Heidler, H.Y. Lim, A. Haussler, B. Albuquerque, B. Zimmer, C. Moser, C. Behrends, F. Koentgen, I. Wittig, M.H. Schmidt, A.M. Clement, T. Deller, I. Tegeder, 2016) [1].

**Specifications Table**

**Table:** 

Subject area	*Cell biology*
More specific subject area	*Protein interactions*
Type of data	*Tables*
How data was acquired	*Yeast-2-Hybrid screen with human full-length progranulin as bait against human fetal brain and human bone marrow libraries.*
Data format	Table 1: *Raw Y2H*
	Table 2: *Sorted Y2H according to selection criteria*
	Tables 3, 4: *Gene ontology over-representation analysis*
Experimental factors	*Full length human progranulin was used as bait*
Experimental features	*Matchmaker Y2H technology*
Data source location	*Frankfurt, Germany*
Data accessibility	*Data is with this article*

**Value of the data**

• Progranulin Y2H interactors are useful for comparison of interactors obtained with different methods or with different libraries.
• Progranulin interactors give insight into its putative functions.
• Progranulin Y2H results can be used to build protein networks and signaling paths.

## 1 Data

The full Y2H table contains all of the information and data from the screen and the blast analysis, as well as the sequence of the inserts. If the blast analysis gave two identically scoring hits for a prey sequence, both hits can be found in the full table. A description of the columns is given in comments of the column headers (full table) and explained in the "Info" and in the "Selection Criteria" tables, which are part of the Excel file.

The short table shows a summary of the results sorted according to the libraries (brain and bone marrow) and marked with different colors to indicate likely positive hits (green), putative hits (white), possibly wrong hits (yellow) and false hits (red), based on the selection criteria explained in the "Selection Criteria" table, which are according to recommended standards [2], [3].

The tables "GO terms" and "GO slim" contain the results of the Gene Ontology over-representation analysis of likely positive and putative preys.

## 2 Experimental design, materials and methods

### 2.1 Yeast-2-Hybrid

The Y2H screen was done for human fetal brain and bone marrow libraries with the Matchmaker GAL4 system (Clontech) by ImaGenes GmbH (Berlin, Germany) using Gateway Invitrogen plasmids for human full length progranulin as bait as described in [1]. Preys were categorized according to established selection criteria [2] as described in [1].

### 2.2 Gene ontology

The gene ontology terms associated with the Y2H progranulin-preys were obtained by means of over-representation analysis (ORA) using the web-based GeneTrail tool (http://genetrail.bioinf.uni-sb.de/) [4] as described in [1]. Protein IDs were transformed to Entrez Gene IDs using the ID conversion tool of DAVID 6.7 (Database for Annotation, Visualization and Integrated Discovery; https://david.ncifcrf.gov/home.jsp) and subsequently IDs were submitted to ORA using all genes in the database as background. ORA parameters were set to *p*-value threshold *tp*=0.05 and Bonferroni α correction (Results in Table 3, “GO terms”). In addition, we used the GO slim tool of Panther (Protein ANalysis THrough Evolutionary Relationships; http://www.pantherdb.org/). The results are in Table 4 “GO slim”.
