# Supplementary material for: Yeast-2-Hybrid data file showing progranulin interactions in human fetal brain and bone marrow libraries
Source: Data Brief. 2016 Nov 19;9:1060–2. doi: 10.1016/j.dib.2016.11.031 (PMC5126125; doi:10.1016/j.dib.2016.11.031)

Conflicts of Interest Statement

Manuscript title: Yeast-2-Hybrid data file showing progranulin interactions in human fetal brain and bone marrow libraries

The authors whose names are listed immediately below certify that they have NO affiliations with or involvement in any organization or entity with any financial interest (such as honoraria; educational grants; participation in speakers' bureaus; membership, employment, consultancies, stock ownership, or other equity interest; and expert testimony or patent-licensing arrangements), or non-financial interest (such as personal or professional relationships, affiliations, knowledge or beliefs) in the subject matter or materials discussed in this manuscript.

Author names:

Irmgard Tegeder

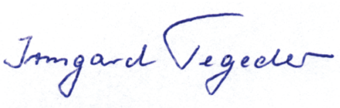

Supplement: Supplementary file 1 — Supplementary material [file mmc1.pdf]
